# Sequential mixed method evaluation of the acceptability, feasibility, and appropriateness of cognitive behavioral therapy for psychosis stepped care

**DOI:** 10.1186/s12913-022-08725-5

**Published:** 2022-11-05

**Authors:** Sarah L. Kopelovich, Jessica Maura, Jennifer Blank, Gloria Lockwood

**Affiliations:** 1 Department of Psychiatry and Behavioral Sciences, Harborview Medical Center, University of Washington School of Medicine, 325 Ninth Avenue, Box 359911, Seattle, WA 98104 USA; 2 Harborview Medical Center, University of Washington School of Medicine, Seattle, WA USA

**Keywords:** Cognitive behavioral therapy for psychosis, Stepped care, Community mental health, Implementation

## Abstract

**Background:**

Cognitive Behavioral Therapy for psychosis (CBTp) is recommended by national treatment guidelines yet remains widely inaccessible in the U.S. A stepped care model, favored and feasible for other scarce interventions, may improve access to CBTp.

**Methods:**

We employed an exploratory sequential mixed method design inclusive of two distinct phases to quantitatively evaluate the acceptability, feasibility, and appropriateness of CBTp Stepped Care (CBTp-SC) among practitioners who were trained in low-intensity CBTp (Step 1), Group-Administered CBTp (Step 2), and Formulation-based CBTp (Step 3). In Phase 1, we queried respondents using the Acceptability of Intervention Measure, Intervention Appropriateness Measure, and the Feasibility of Intervention Measure to ascertain perceptions of these leading indicators of implementation success. In Phase 2, we conducted focus groups with CBTp-SC-trained practitioners (*n* = 10) and administrators (*n* = 2) from 2 of the 4 Phase 1 study sites to evaluate the theoretical assumptions of stepped care and to better understand key barriers and facilitators.

**Results:**

Forty-six practitioners trained in all three levels of CBTp-SC completed the online survey in Phase 1. All participants were employed by a community mental health agency currently sustaining CBTp-SC. Respondents endorsed high levels of acceptability, feasibility, and appropriateness for the CBTp-SC model. We found evidence to suggest that licensed practitioners and Step 3 practitioners perceived formulation-based CBTp as more appropriate for their clients. In Phase 2, six themes emerged which affirmed the utility of the model for stakeholders, supported stepped care theoretical assumptions, and revealed key areas for improvement.

**Conclusions:**

Early adopters of CBTp-SC in the U.S. perceive it to be acceptable, feasible, and appropriate in community mental health care settings. Practitioners and administrators identified training and implementation barriers, including the importance of organizational readiness, a CBTp coordinator role, and a desire to adapt the intervention. These early findings will facilitate iterative refinement of the stepped care model for U.S. public behavioral health agencies. Additional research is needed to explore perceptions and clinical outcomes among CBTp service users.

## Background

Cognitive behavioral therapy for psychosis (CBTp) is an evidence-based psychotherapeutic treatment that targets the distress or functional impairment related to psychotic symptoms. Despite evidence supporting the efficacy of CBTp [1], recent point prevalence estimates suggest a ratio of 15 CBTp-trained practitioners for every 10,000 Americans with a psychotic disorder [2]. The United Kingdom, which also suffers from poor rates of CBTp access, adopted a stepped care approach to psychological treatment delivery in an attempt to redress inaccessibility of evidence-based treatments for serious mental illness (SMI). The Improving Access to Psychological Therapies for SMI (IAPT-SMI [3]) represents one of the broadest systemic reforms to care delivery to address the persistent gap between schizophrenia treatment guidelines and schizophrenia treatment delivery. IAPT employs a stepped care service delivery model. Stepped care is a system of delivering and monitoring treatments so that the most effective, yet least resource-intensive intervention is delivered to clients [4]. This model involves an initial assessment to identify the least intensive treatment required to best meet an individual’s needs and monitoring treatment outcomes to determine whether the client has been assigned to the appropriate level of care and making dynamic data-based decisions about whether a service user should be ‘stepped up’ to more intensive care or ‘stepped down’ to less intensive care, as needed. Bower and Gilbody [5] identified cognitive behavioral therapy as a prime candidate for stepped care service delivery for three key reasons: its prevalent inclusion in national treatment guidelines, its core theoretical model, and common therapeutic techniques across principle- and protocol-based cognitive behavioral therapies.

Kopelovich and colleagues [6] conceptualized CBTp stepped care as both an implementation and service delivery model based on three core principles: (1) stepped care decisions are predicated on structured professional judgment and shared decision-making; (2) levels of CBTp treatment are discrete; and (3) CBTp-SC is responsive to individual and organizational needs and preferences while maintaining adherence to a prescribed structure. The authors proposed a model with three discrete levels of CBTp care: Step 1 consists of low-intensity cognitive behavioral techniques for psychosis (LI-CBTp, for example, CBTp-informed techniques integrated into case management sessions), Step 2 consists of group-administered CBTp, and Step 3 consists of high-intensity CBTp (e.g., individual formulation-based CBTp). As an implementation model, stepped care holds appeal for maximizing an understaffed workforce by enabling training of a broader array of behavioral health, allied, and non-professionals in applications of CBT that are concordant with their credential, role, and scope of practice. As a service delivery model, a stepped care approach can enhance the efficiency with which service users are able to access the intervention (efficiency) while maintaining a right fit for their clinical needs (equivalence) [5].

Empirical evidence is amassing on the efficacy of interventions that may be considered in a CBTp-SC implementation. The largest evidence exists for formulation-based individual CBTp (Step 3), with evidence of small-moderate effects on positive symptoms [7–9] and negative symptoms [7]. Group CBTp (Step 2) has also received empirical attention but has not been studied as extensively as individual CBTp. Roughly 10 randomized controlled trials (RCT) and 7 smaller studies (pre-post or randomized) have been published that evaluate the efficacy of group CBTp for decreasing psychotic symptoms or symptom-related distress compared to treatment as usual (e.g., [10]) and—to a lesser extent—an active comparator (e.g., [11]). In a comprehensive analysis of extant RCTs, Wykes and colleagues [10] found no evidence of a statistically significant difference in effect size between individual and group CBTp for the target symptom (individual ES = 0.415; group ES = 0.386). Multiple studies have found evidence that group CBTp enhances coping, self-esteem, and functioning against both active and inactive comparators [11–21] and may therefore serve to help individuals better tolerate residual psychotic symptoms. Consensus definition of low-intensity CBTp (Step 1) has been elusive. However, consensus concerning core characteristics of LI-CBT has emerged [22] from the 30 systematic reviews and 50 controlled trials supporting the effectiveness of LI-CBT across a range of mental health conditions [23, 24]. A recent systematic review and meta-analysis on LI-CBT for psychosis demonstrated significant between group effects on symptoms of psychosis (*d* = -0.46) that were sustained at follow-up (*d* = -0.40) across the 10 controlled trials that met inclusion criteria [25]. The authors concluded that “overall, findings suggest that low intensity CBTp shows promise with effect sizes comparable to those found in meta-analyses of CBTp more broadly. We suggest that low intensity CBTp could help widen access” (p.183). Although LI-CBTp interventions included in Hazell and colleague’s meta-analysis [17] all relied upon practitioners with a formal psychological therapy qualification, other studies have demonstrated the feasibility, acceptability, and effectiveness of non-therapist administration of LI-CBTp interventions [26–32]. In practice, non-therapist practitioners are commonly trained as LI interventionists [27, 33, 34] and are critical to the implementation of the CBTp-SC model [6].

While stepped care has intuitive appeal to address the critical shortage of trained CBTp practitioners, to-date there has been no published evaluation of CBTp stepped care (CBTp-SC). This article represents the first evaluation of the U.S.-adapted CBTp-SC model and aims to explore the fundamental assumptions that CBTp-SC is feasible, acceptable, and appropriate for community behavioral health settings in the U.S.; to ascertain whether significant differences emerge in the challenges associated with learning or perceived usefulness and applicability of the different CBTp interventions; and to explore the perceptions of equivalence and efficiency among a sample of CBTp-SC practitioners and administrators settings that are currently sustaining the model.

## Study methods

We conducted two discrete but complementary studies following an exploratory sequential mixed method design, which entails collecting and analyzing quantitative and then qualitative data in two consecutive phases within one study [35]. This form of mixed methodology is considered a popular but onerous methodology to employ. We adhered to the recommendations in executing and reporting a sequential mixed method study outlined by Ivankova and colleagues [36]. Phase 1 consisted of a cross-sectional survey of CBTp-SC practitioners; the primary aim was to quantitatively evaluate the extent to which CBTp-trained practitioners across the population of CBTp-SC sites endorsed the acceptability, appropriateness, and feasibility of the model. Phase 2 intended to further explore these perceptions and better grasp both their sources and impacts on sustainment.by applying a qualitative approach among two of the CBTp-SC sites included in Phase 1. This study was reviewed by the Washington State Institutional Review Board and was granted exempt determination. All procedures were carried out in accordance with relevant guidelines and regulations. Participants provided informed consent to participating in study activities.

### Population

We sampled four multi-site community mental health agencies (CMHA) that were all in the sustainment phase of CBTp-SC in the U.S. At the time of data collection (Winter 2019), this sample constituted the population of CBTp-SC sites in the U.S. At each site, the University of Washington CBTp implementation team worked with CMHA leadership to prepare for systematic implementation of CBTp over an 18-month period from pre-implementation to sustainment phase. Behavioral health and allied practitioners were allocated to a CBTp level of care based on credentials, qualifications, and the nature and scope of their role within the agency. Levels of care were consistent with those prescribed by Kopelovich and colleagues [6], as were decisions pertaining to the allocation of agency practitioners to level of care training (e.g., credentials, qualifications, role, and scope of practice).

### Training

All trainees received longitudinal support over a 12-month period using a multimodal training approach. Once the decision was made by agency leadership to implement CBTp, prospective trainees were invited to an information session intended to orient them to the initiative, training requirements, and to enhance motivation to participate. An orientation packet also described the elements of each track’s training program, training calendar, and requirements for certificates of completion. All enrolled practitioners received pre-workshop materials (e.g., CBTp texts, access to a self-paced CBTp foundational e-training) and met together on workshop day 1 to receive didactic and experiential training in cognitive behavioral theory, cognitive theory of psychotic symptoms, CBTp evidence base, an orientation to CBTp-SC, and guided rehearsal of clinical assessments to facilitate the CBTp-SC referral and assessment process. Subsequently, practitioners were divided into training tracks in which they met with the lead trainer for an additional 14 h. All workshops included didactic presentation of the CBT conceptualization of psychosis, phases of treatment, and brief review of the evidence base for their training protocol. Experiential activities included empathy exercises, live and/or recorded demonstrations, role plays with feedback, and applied learning through case studies. Subsequent to the workshops, all practitioners engaged in longitudinal training consisting of biweekly clinical case consultation, didactics, and case-based role plays for 6–12 months. Finally, practitioners received individual feedback on performance samples (e.g., role plays, audio sessions).

#### Phase 1: quantitative analysis of CBTp stepped care

##### Sampling and recruitment

Eligible participants included any provider who partook in the CBTp-SC training program conducted by the University of Washington CBTp-SC implementation team in the past 5 years (*N* = 112). Participants consisted of healthcare professionals and allied professionals aged 18 years and older employed by one of the four community behavioral health agencies in which CBTp-SC was implemented. Prospective participants were sent an initial email describing the research along with a unique link to the web-based questionnaire. Respondents received a $15 electronic gift card as compensation for participation.

##### Measures

The web-based questionnaire consisted of 30 items, inclusive of participant demographics and professional background, time since CBTp-SC training, and level of care trained in (e.g., Step 1, Step 2, Step 3). Participants completed three 4-item measures intended to assess their perceptions of the acceptability, feasibility, and appropriateness of the CBTp-SC model to their patients. *Acceptability*, which is defined as the perception among implementation stakeholders that a given practice or innovation is agreeable, palatable, or satisfactory, was measured by the Acceptability of Intervention Measure (AIM [37]). *Feasibility*, defined as the extent to which a practice or innovation can be successfully used or carried out within a given agency or setting, was measured by the Feasibility of Intervention Measure (FIM [37]). *Appropriateness* is defined as the perceived fit, relevance, or compatibility of the innovation or evidence-based practice for a given practice setting, provider, or consumer, and was measured with the Intervention Appropriateness Measure (IAM [37]). These three constructs are considered leading indicators of implementation success [38]. The AIM, FIM, and IAM have demonstrated acceptable content validity, discriminant validity, reliability, structural validity, and responsiveness to change [37]. Each measure consists of four items on a 5-point Likert scale (1 = completely disagree, 2 = disagree, 3 = neither agree nor disagree, 4 = agree, 5 = completely agree). Three additional attitudinal questions developed by the investigators were added to the survey in an attempt to gain additional insights in the perceived *usefulness* (“How useful is the application of the CBTp protocol in which you were trained to your patients experiencing psychosis?*”*), *applicability* (“How well do the components of the CBTp protocol you were trained in address the problems/conditions of the patients you delivered the treatment to?”), as well as *challenges* with administering CBTp-SC (“How challenging was applying the CBTp model to your patients?*”*) on a 5-point Likert scale (1 = not at all, 2 = a little bit, 3 = moderately, 4 = quite a bit, 5 = extremely).

##### Data analysis

Data were collected and managed using REDCap (Research Electronic Data Capture [39]), a secure, web-based software platform. Prior to running analyses, all data were de-identified and screened for outliers and missing data. Although we found evidence that our data across variables were non-normally distributed based on Shapiro-Wilks’ tests, we conducted one-way analysis of variance (ANOVA) tests due to the test’s robustness for data that deviate from normal [40] in addition to descriptive statistics of our samples as a whole and within respective steps. Statistical significance was defined as *p*-values less than 0.05. As an alternative to post-hoc power analysis, analysis of the width and magnitude of the 95% confidence interval was selected as the more appropriate method of determining statistical power [41].

##### Results

All 112 individuals who had previously been trained in CBTp Step 1, 2, or 3 at each of the four CBTp-SC sites were invited via email to participate in the online questionnaire. Thirty recruitment emails were returned due to non-working emails. Forty-seven participants consented to participate (response rate = 57.3%). Removing one questionnaire due to missing data yielded an analytic sample of 46. Table 1 reports demographics and professional characteristics of the final sample. Roughly the same number of participants were trained in Step 1 (57%), Step 2 (46%), and Step 3 CBTp (57%), with 35% trained in two levels of care, and 11% trained in all three levels of care. Respondents self-selected the level of care they predominantly provide. Sixteen participants (35%) primarily administer Step 1, 10 participants (22%) administer Step 2, and 20 participants (44%) administer Step 3. Most respondents (80.4%) reported they were currently administering CBTp to clients at the time they completed the survey. Because each respondent had administered CBTp-SC at some point following training, we included data from each of the 46 respondents in our analyses, even if they indicated they were not currently providing CBTp-SC. On average across all levels of care, respondents estimated that between 15–16 of the clients on their caseload were eligible for CBTp and reported providing the intervention to 10 clients.

**Table 1:** Demographics and Professional Characteristics

Demographics	**Mean (SD)**	
Age	46.83 (11.6)	
Gender	**N**	**%**
Man	16	34.8
Woman	28	60.9
Non-binary	1	2.2
Discipline		
MHP Unlicensed	11	23.9
MHP Licensed	30	65.2
Peer Support Specialist	5	10.9
Professional Characteristics	**Mean (SD)**	
Years of clinical experience	10.89 (8.7)	
Time since first CBTp training	**N**	**%**
6–11 months ago	3	6.5
1–5 years ago	39	84.8
More than 5 years ago	4	8.7
Ongoing professional development in CBTp^a^		
Not receiving any professional development	3	6.5
Group consultation through agency	27	58.7
Individual consultation through agency	14	30.4
CBTp ECHO Clinics	21	45.7
Other (Did not specify)	5	10.9

^a^ Participants were able to select more than one option

The primary findings for Phase 1 reveal that respondents across all levels of care reported high mean scores of acceptability, appropriateness, and feasibility (Table 2). Respondents indicated higher than moderate scores on perceived usefulness of CBTp (*M* = 3.87, *SD* = 0.94), found the CBTp model to be moderately challenging to apply with their patients (*M* = 3.14, *SD* = 0.96), and reported a moderate degree of fit for the CBTp step they administered to their client’s problems (*M* = 3.51, *SD* = 0.84). A summary of these outcome variables across the primary level of care provided can be found in Table 2.

**Table 2:** Summary of Outcome Data

	Step 1 (*n* = 16)	Step 2 (*n* = 10)	Step 3 (*n* = 20)	All responders (*N* = 46)		
					95% CI	
	Mean (SD)				LL	UL
**Acceptability of Intervention Measure (AIM)**						
CBTp-SC meets my approval	4.31 (.70)	4.30 (.67)	4.60 (.60)	4.43 (.65)	4.24	4.63
CBTp-SC is appealing to me	4.00 (.89)	4.25 (.79)	4.70 (.73)	4.36 (.85)	4.11	4.61
I like CBTp-SC	4.06 (.68)	4.30 (.67)	4.58 (.84)	4.33 (.77)	4.10	4.56
I welcome CBTp-SC	4.13 (.72)	4.50 (.53)	4.75 (.44)	4.48 (.62)	4.29	4.66
AIM Total Mean Score	4.13 (.68)	4.34 (.62)	4.64 (.62)	4.39 (.67)	4.19	4.59
**Intervention Appropriateness Measure (IAM)**						
CBTp-SC seems fitting	4.25 (.58)	4.20 (.92)	4.55 (.51)	4.37 (.65)	4.18	4.56
CBTp-SC seems suitable	4.25 (.58)	4.40 (.52)	4.50 (.51)	4.39 (.54)	4.23	4.55
CBTp-SC seems applicable	4.25 (.58)	4.20 (.92)	4.55 (.51)	4.37 (.65)	4.18	4.56
CBTp-SC seems like a good match	4.25 (.58)	4.10 (.88)	4.50 (.69)	4.33 (.70)	4.12	4.53
IAM Total Mean Score	4.25 (.58)	4.23 (.77)	4.53 (.53)	4.36 (.60)	4.18	4.54
**Feasibility of Intervention Measure (FIM)**						
CBTp-SC seems implementable	4.19 (.54)	3.90 (.99)	4.35 (.59)	4.20 (.69)	3.99	4.40
CBTp-SC seems possible	4.25 (.58)	4.40 (.70)	4.55 (.51)	4.41 (.58)	4.24	4.59
CBTp-SC seems doable	4.13 (.50)	4.20 (.63)	4.50 (.51)	4.30 (.55)	4.14	4.47
CBTp-SC seems easy to use	3.81 (.83)	3.90 (.99)	3.70 (.92)	3.78 (.89)	3.52	4.05
FIM Total Mean Score	4.09 (.53)	4.10 (.73)	4.28 (.58)	4.17 (.59)	4.00	4.35
**Experience Using the CBTp Model**						
How useful is the application of the CBT protocol in which you were trained to your patients experiencing psychotic symptoms?	3.50 (1.03)	3.78 (1.09)	4.20 (.70)	3.87 (.94)	3.58	4.15
How challenging was applying the CBTp model to your patients?	3.31 (1.08)	2.89 (1.05)	3.13 (.83)	3.14 (.96)	2.86	3.43
How well do the components of the CBTp protocol you were trained in address the problems/conditions of the patients you delivered the treatment to?	3.31 (.87)	3.11 (.78)	3.85 (.75)	3.51 (.84)	3.26	3.76

We used a one-way ANOVA to examine variations in our outcome variables across discipline and level of care. Results can be found in Table 3. Homogeneity of variance was not violated for any of the ANOVAs (*p* > 0.05). No significant differences were found between levels of care for the AIM, IAM, or FIM. However, the ANOVA *F*-test detected a significant difference by discipline for the applicability of CBTp question, “*How well do the components of the CBTp protocol you were trained in address the problems/conditions of the patients you delivered the treatment to?*” Licensed mental health practitioners were significantly more likely than unlicensed mental health practitioners to endorse applicability of CBTp to their patients. Post hoc analysis with a Bonferroni adjustment were applied to compare differences across levels of care; a significant difference was detected in perceptions of applicability; further analysis revealed a trend toward significance resulting from Step 3 practitioners endorsing higher perceptions of applicability.

**Table 3:** Acceptability, appropriateness, and feasibility compared across discipline and primary level of care

Source	df	SS	Mean sq	F	*p*-value
**Discipline**					
AIM x Total Score	2, 42	12.65	6.32	0.87	.43
IAM x Total Score	2, 43	14.52	7.26	1.25	0.30
FIM x Total Score	2, 43	7.84	3.92	0.69	0.51
Usefulness	2,42	3.86	1.93	2.29	0.11
Challenging	2,42	0.81	0.40	0.43	0.65
Applicable	2,42	7.02	3.51	6.08	0.005**
**Primary Level of Care Provided**					
AIM Total Score	2, 42	38.14	19.07	2.88	0.07
IAM Total Score	2, 43	14.71	7.36	1.27	0.29
FIM Total Score	2, 43	5.79	2.90	0.51	0.61
Usefulness	2,42	4.44	2.22	2.69	0.08
Challenging	2,42	1.05	0.52	0.56	0.58
Applicable	2,42	4.34	2.18	3.41	0.04*

* *p* ≤ .05

** *p* ≤ .005

#### Phase 2: qualitative analysis of CBTp stepped care

Phase 2 set out to use qualitative methods to further explore perceptions of CBTp-SC among two groups of stakeholders: practitioners who were trained in the intervention and administrators at implementation sites. Focus groups and interviews were selected as the primary source of data collection for the qualitative analysis as they provide some structure through investigator prompts while also allowing for free-flowing and interactive responses. These methods were deemed conducive to developing a comprehensive understanding of the perceived equivalence, efficiency, and acceptability of the CBTp-SC model.

##### Sampling and recruitment

The two of the four CMHAs that had implemented CBTp-SC for the longest duration were purposively recruited. The administrators who supervised CBTp at their agencies and practitioners who enrolled in the CBTp-SC program were invited to participate in the focus groups. Clients who engaged in treatment with a CBTp-SC provider were also intended to be involved in focus groups. However, safety and logistical concerns prompted by the COVID-19 pandemic required an indefinite deferral of the service user interviews. Administrators and clinicians responded to an email invitation sent by a member of the study team (blinded), which asked them to take part in a focus group to learn more about the successes and challenges of implementing CBTp-SC. All participants were provided with a $25 electronic gift card following their participation.

##### Focus groups/interviews

The study team developed an interview guide, which aimed to assess perceptions of the equivalence (e.g., *“How do you feel about your ability to improve functional recovery for clients using a CBTp-SC model compared to treatment as usual?”*), efficiency (e.g., *“In your experience, how effective was the CBTp-SC model at reducing the wait time for clients to access some form of CBTp?”*), acceptability (e.g., *“How do other practitioners/leaders in your setting view CBTp-SC?”*), and feasibility (e.g., “What problems arose in how CBTp-SC was integrated into your clinic?”) of the CBTp-SC model among both sets of participants. Administrator interviews focused on perspectives of how CBTp-SC was implemented and the therapeutic impact of the CBTp-SC program in their clinic. Practitioner focus groups focused primarily on impressions of the process of adapting, adopting, and integrating CBTp-SC into the agency workflow, including successes and challenges associated with implementation. One administrator interview was conducted face-to-face, as this interview occurred just prior to the onset of the COVID-19 pandemic. This interview lasted approximately 60 min. All other interviews and focus groups were conducted via videoconferencing and lasted either 60 min (administrative interview) or 90 min (provider focus groups).

Focus groups and interviews were facilitated by a Clinical Psychology Postdoctoral Fellow and co-facilitated by a Research Coordinator, neither of which were involved in training or implementation activities for the enrolled participants. All interviews and focus groups were audio recorded (with all participants’ consent) and professionally transcribed. Following transcription, transcripts were compared to audio recordings in order to correct missing and/or unintelligible data, all identifying information was removed from transcripts, and data was collated to result in one administrator transcript and one clinician transcript.

##### Analysis

The qualitative data was analyzed by the Clinical Psychology Postdoctoral Fellow and Research Coordinator who facilitated the focus groups/interviews. Thematic analysis was conducted as outlined by Braun and Clarke [42]. This involved a phase of pre-coding in which both coders independently reviewed transcripts and noted initial thoughts and patterns, followed by a line-by-line analysis of the transcripts in order to develop initial codes. Although initial codes were generated from the data, data was interpreted within the context of the research question, which revolved around the equivalence, efficiency, and acceptability of the CBTp-SC model. Initial codes were developed independently by both coders beginning with one transcript. Following this initial coding session, both coders met to engage in a peer debriefing session to discuss separately identified codes, review discrepancies in how data was initially coded, and begin to establish a working code book. This procedure of independent coding, peer debriefing, and ongoing development of the code book was then repeated for the second transcript. At this time, both coders independently collated codes into potential themes, followed again by a period of peer debriefing, review of discrepancies in themes, and collaborative theme development. Both coders then collaboratively reviewed themes to ensure that identified themes were coherent in relation to individual coded extracts and the entire dataset. This review led to final adaptations to the core thematic structure of the data, the defining and naming of themes, and the production of the report.

## Results

### Participant characteristics

Two administrators (one from each participating agency) and 10 clinicians (five from each participating agency) consented to participate. Table 4 provides descriptive data of all 12 participants who completed the interviews/focus groups. Of the 10 clinicians who engaged in the focus groups, 70% were female and 90% had obtained a Master’s degree in either Social Work or Psychology. Eighty percent identified as White, one individual identified as Black, and one individual preferred not to respond to this question. The mean age of clinicians was 39 years old (*SD* = 7.96). All clinicians reported being trained between 4–6 years ago. Our sample consisted of respondents who were providing all three levels of care, including Step 3 (60%), Step 2 (10%), and Step 1 (10%), as well as two clinicians who had been trained to administer two levels of care (20%). Administrators who engaged in interviews were female, identified as white, and had obtained a Master’s degree in Social Work (100%). Their mean age was 47 years old (*SD* = 4.24)*.*

**Table 4:** Socio-demographic characteristics of focus group/interview participants (*n* = 12)

ID	Gender	Degree	Training Received	Year Trained
**C1**	Female	MSW	Step 3	2016
**C2**	Female	MA	Step 3	2015
**C3**	Male	MA	Step 3 and Step 2	2016 2017
**C4**	Female	MSW	Step 3 and Step 1	2016 2017
**C5**	Female	MSW	Step 3	2015
**C6**	Male	BA	Step 1	2017
**C7**	Female	MA	Step 3	2017
**C8**	Male	MA	Step 2	2017
**C9**	Female	MA	Step 3	2017
**C10**	Female	MSW	Step 3	2017
**A1**	Female	MSW	–	–
**A2**	Female	MSW	–	–

#### Key themes

The data from the focus groups were organized into six key themes: (1) previous clinical approaches to psychosis; (2) alignment with values; (3) fit of the model; (4) consultation benefits; (5) organizational readiness; and (6) implementation needs.

##### Theme 1: enhanced clinical approaches to psychosis

Before implementation of the CBTp-SC model, treatment for individuals with psychosis at participating agencies largely consisted of case management and informal uses of components of evidence-based practices, such as CBT or Motivational Interviewing (A1: *“…prior to [implementing CBTp-SC], the clinical model was really just kind of your classic case management with maybe some basic CBT skills included but nothing formalized. Case managers were all-encompassing prior to that…they did everything with their interdisciplinary team but they were the primary care coordinator”*). Similar sentiments were expressed by clinicians, who described an emphasis on medication compliance and supportive therapy (C9: *“Meeting with clients with psychosis, it was a lot about [medication] compliance… There was a lot of case management back then instead of meeting the client where they're at and helping them manage the distress”*). The historical emphasis on case management combined with an unstructured treatment approach to target psychotic symptoms contributed to a sense of disengagement from their work with these populations (C5: *“In terms of working with people with psychosis, I don't think that I gave as much, I don't want to say energy, but I think I wasn't as thoughtful about how to, I guess, deliver interventions and treatment”*), as well as avoidance of discussions of psychotic experiences (C2: *“ I didn't really want to get into the nitty-gritties of the delusions or the voices so much. I wanted to acknowledge that they were there and validate it… but I didn't talk specifics.”*). There was unanimous agreement among clinicians and administrators that CBTp-SC enhanced access to CBTp.

##### Theme 2: CBTp-SC aligned with organizational and individual values

For both administrators and clinicians, the CBT-SC model served as a means to establish evidence-based practices that would meet the needs of the clinic population (A1: *“We had an evidence-based practice steering committee that was developed…wanting to look at various evidence-based modalities that could be implemented. CBTp was one of them that I think we found would be well-suited to the population that we serve”*). Clinicians viewed CBTp-SC as an opportunity to improve proficiency (C9: *“A lot of my caseload was people who were diagnosed with schizophrenia spectrum disorder. I really felt inadequate in how to provide services to them. I was very excited that there would be some training on how to provide therapy for most of my caseload”*) and confidence (C2: *“It was just a population that I probably felt the least confident in working with and so, I really wanted to learn to better serve that group”*) in working with individuals with psychotic spectrum disorders. A concern was also expressed around the potential consequences of not delivering agency-promoted interventions (C3: *“I had a fear that I would not be allowed to do therapy unless I was trained in some of these things, which– I guess that was part of the impetus for me”*). In addition to overt and perceived pressures to engage in training, clinicians also reported that clinical supervisors offered incentives to clinicians engaged in CBTp-SC, including reduced caseloads and protected time for session planning and ongoing training.

##### Theme 3: organizational readiness for CBTp-SC

Respondents highlighted a number of challenges that occurred as CBTp-SC was first implemented at their agencies. Both administrators and clinicians reported limited understanding of the model at the outset (C5: *“I don't think I understood anything about the different steps before attending the training*”) that was gradually remediated over the course of the pre- and peri-implementation phases. Relatedly, administrators disclosed a lack of pre-defined organizational processes outlining how the CBTp-SC model would be incorporated into established clinic policies and practices (A1: *“I think for us the challenge lies in the operational workflows and processes that need to be set up. Essentially just having organized understanding of who's trained in which modality. Who has space to see new patients? Having someone who's dedicated to be able to organize that and keep track of that within the clinic [is needed]”).* Limited organizational preparation consequently impacted the effective application of structured referral procedures (A2: “*We don't have a mechanism in place for our access to care team to do a screening and match people to trained clinicians*”) and access to the intervention (C1: “*[Barriers to getting other practitioners to refer] was frustrating when one of us had openings and was trying to get someone in when we know how many people might actually benefit from it*”).

##### Theme 4: fit of the model

Broadly, respondents indicated that CBTp-SC was highly relevant to their clinic populations (C3: “*Everyone has someone on their caseload who meets this criteria*”) and viewed the program as an opportunity for clinicians to obtain specialized training and clinical expertise in the treatment of psychotic spectrum disorders (A1: *“Benefits were obviously the training and clinical expertise that it brought to staff…I think it's strengthened our workforce and brought a critical skill to the people who attended the training”*). The fit of the CBTp-SC model was also demonstrated by the reported benefits to both clients (A2: “*…reduced decompensation, reduced hospitalization, a reduced need to use crisis resources like our ENTs and stabilization, increased insight, decreased distress as a result of symptoms, and increased level of functioning and engaging in life goals that aren't related to treatment, like employment and relationships and family and education*”) and clinicians (A2: “*All of the clinicians…they're so proud that they're trained in this, they're so proud that we offer this, and it's really appealing to them that they feel that they're offering this amazing evidence-based practice that people in private insurance are not able to receive because it's pretty scarce in our area*”).

Despite the apparent fit of the model, clinicians and executives described a number of concerns with the ability to use the CBTp-SC model to fidelity. Notably, respondents described concern with how to implement this structured model into community mental health or assertive community treatment teams, both of which often require flexible approaches to referrals, scheduling, and intervention delivery (C7: “*I really wanted to implement it… but there's anxiety associated with taking the ideal version of it and implementing it into the community mental health agency*”). Consequently, respondents described incidences in which the model was adapted in order to meet the needs of the agency, such as loosening enrollment criteria (C6: “*I can say that for the groups, we're in a smaller branch, so you have to have a certain number of participants to have a group. We had to take in enough people that it became just a CBT group*”) or informally utilizing components of the intervention (A2: *“[CBTp-SC has] certainly given [clinicians] tools to use when they work with somebody, but I don't think that they have the ability to use it to fidelity*”).

##### Theme 5: consultation benefits

The external consultation model provided through CBTp-SC was considered to be a significant contributor to skill building and maintenance post-training (A1: “*People go to training all the time, but usually there's really nothing that's helping them to maintain their skills and also get expert consultation to determine if they're delivering the service properly. That was a real value added to the clinic and to the patients*”). Clinicians reported numerous benefits associated with consultation calls, including the accessibility of experts to aid in ongoing skill building (C9: *We can join at any time a consultation group and get our skills. We can refresh our skills and ask questions and increase our experience and knowledge through the consultations*”), as well as opportunities to learn from peers (C1: “*I think it's really helpful getting feedback from clinicians at other agencies, as well as our own, just to add a little richness to the discussion*”) and normalize challenges (C3: “*It's certainly normalizing at times…like, ‘Oh, God, that person's really struggling.’ I don't know, [it’s] sometimes somewhat validating*”).

The absence of protected time for clinicians’ ongoing training and consultation emerged as a significant barrier to reaping the benefits of the longitudinal training model. Clinicians described challenges with balancing the requirements of their position with time for training activities (C1: “*I would generally join if I wasn't being called to other meetings or have conflicts. Also, energy's waning a little bit in the clinic…the few of us that are trained up, we’re squeezing them in where we're able to, but it also can feel a little bit of a strain*”). Consequently, clinicians described the added need for intra-agency clinical consultations, which may facilitate sustainment (C7: “*I think…just being able to come together as clinicians who understand the day to day and how our policies work…and the barriers that we face just at our agency and who we serve… and allowed time for that instead of just trying to carve out time randomly when you want to staff something…that would be helpful in addition to the support we get from [CBTp-SC consultation calls]*”).

##### Theme 6: implementation needs

Many of the observed barriers to training and consultation noted above were also identified as implementation needs by practitioners and leadership. Most notably, deficiencies in organizational structure were identified as a primary challenge to implementation. A potential solution to this challenge offered by leadership was the identification of a dedicated point person to manage recruitment, case assignment, determine level of need, and train relevant staff on referral procedures (A2: “*If there was somebody whose position was exclusively the CBTp coordinator in an agency and could market to the community the service that's provided…and…If we had a mechanism in place that could use the screening instrument to identify which clinician and which step would be best, I think that that would be phenomenal and certainly could be something that could be financially beneficial to the agency*”). Relatedly, respondents identified the need for general agency onboarding to the CBTp-SC model to facilitate referrals (C3: “*It almost felt like there needed to be a training for the rest of the clinic*”). Respondents also noted insufficient availability of staff trained in each of the three levels of care, which impeded timely stepping up/down of care when indicated and limiting wait times for appropriate care (C7: “*It would be probably more helpful…to have individuals trained in all three tracks because…I was trained in step three and…I couldn't refer people who are more scaled down to step one because there was nobody available to take them for a period of time*”).

A number of potential contributors to these challenges were also discussed by respondents. These included limited resources available within community mental health systems (C1: “*We’re in a managed care system, which just makes it impossible to get the protected time that we would really need. That’s been the biggest challenge for me is just not having the time for seeing people or for training*”) that consequently contributed to feelings of burden amongst both administrators (A2: “*It would have been a full-time job for me to make sure that I was taking into consideration every aspect of what needed to be done*”) and clinicians (C2: “*We couldn't easily block off our schedules. Even if we did, the reality was, we had a crisis going on…It's just the nature of the beast that just didn't easily allow for us to block the time or our managers to really give us the time either*”). A. These tensions were echoed by practitioners when incentives such as protected time and reduced caseloads were not realized during implementation (C2: *“Case managers were delivered a false promise that their caseloads would be reduced if they engaged in an EBP… because they would need this extra protected time to dedicate to learning it and taking on potentially more challenging and time-consuming clients, but that never really came to fruition…”*). Additionally, perceptions surrounding the ability of individuals with psychosis to engage in CBTp-SC were identified by one administrator as a potential challenge to implementation (A1: “*Just the nature of the disease is such that a lot of times those are patients that don't really engage in services to begin with*”).

Leadership support was identified as a core feature of successful implementation (C8: “*Our director was super supportive. I think that without her championing, like the population, just individuals with schizophrenia spectrum as well as the modality itself I don't think…it wouldn't have been implemented at all*”), as was the continued accessibility of trainings from the CBTp-SC implementation team (A1: “*The ability to train new staff coming in has been key to keeping it alive*”). Clinicians who did receive protected time during the training period highlighted this as an implementation facilitator (C10: “*I think just getting more time for additional training and training building [is important]*”). Finally, external (CBTp-SC implementation team) and intra-agency consultation opportunities were again highlighted by clinicians as a core facilitator to foster skill development (C9: “*…consultation is very important to maintain your skills and to continue learning the model. A lot of us who were originally trained with [the CBTp-SC team], we still have access to [those] consultations…our people that are in-house don't get that kind of consultation*”).

## Discussion

CBTp-SC is intended to enhance the accessibility of CBTp, which remains scant despite its robust evidence base and decades-long inclusion in national treatment guidelines. This exploratory sequential mixed method investigation represents the first evaluation of a U.S.-adapted stepped care model among early adopters of this implementation and service delivery model. We sought to assess the extent to which practitioners and administrators perceived CBTp-SC to be acceptable, appropriate, and feasible to implement, as these implementation outcomes are often considered “leading indicators” of implementation success [38]. We relied on qualitative methods to further assess for indicators that CBTp-SC meets the fundamental assumptions of stepped care delineated by Bower and Gilbody [5] and to explore barriers and facilitators identified by CBTp-SC stakeholders at the two longest-standing CBTp-SC agencies in the U.S.

Our findings are generally consistent with prior research regarding attitudes of practitioners trained in CBTp [43, 44] and extend these largely favorable attitudes to the CBTp-SC model. Practitioners across all four CMHAs reported high levels of acceptability, appropriateness, and feasibility of CBTp-SC. We observed no variation in attitudes of CBTp-SC appropriateness, feasibility, acceptability by provider discipline or level of CBTp in which they were trained. Licensed mental health practitioners perceived greater applicability of CBTp to their patients than unlicensed mental health practitioners. Similarly, perceptions of acceptability, feasibility, and appropriateness showed little variability by level of care, with high total mean scores across groups. Clinicians trained in formulation-based CBTp may perceive CBTp-SC as more applicable to their clients than those trained in Step 1 or 2, however the difference merely approached the significance threshold of *p* < 0.05.

Our qualitative analysis identified several important themes that shed light on these preliminary implementation findings and provided support for Bower and Gilbody’s stepped care assumptions. First, respondents felt that CBTp-SC enhanced both access to and quality of care being provided to CMHA patients. In particular, clinicians noted that CBTp implementation resulted in more evidence-based and recovery-oriented practices compared to pre-implementation services. Second, CBTp-SC aligned with organization and individual values. Third, challenges with organizational readiness for CBTp-SC, beginning with simply understanding the implications for policies, practices, and referral and workflows impaired the implementation and administration of CBTp. Fourth, CBTp-SC was determined to be a good fit for CMHA client population as well as for CMHA staff, but multiple respondents commented on adaptations indicated by the service user, the clinician, and the setting. Fifth, stakeholders identified substantial benefit from longitudinal case consultation from experts. That said, clinicians disclosed unmet need for protected time to learn to deliver the intervention during the initial learning period, a preference for shifting to internal clinical consultation following the initial training period, and the need for a CBTp coordinator. Finally, respondents suggested that many of the identified implementation challenges could be remediated with a dedicated CBTp coordinator to manage referrals, case assignments, ensure agency staff are aware of CBTp-SC, and to work with leadership to redress challenges associated with learning or administering the treatment.

Overall, Phase 2 data provided helpful context for understanding perceptions about the usefulness of CBTp as well as challenges in applying CBTp-SC observed in Phase 1. Barriers and facilitators identified in the qualitative phase parallel themes identified in recent U.S. CBTp domestic policy papers advocating for systematic implementation of CBTp in routine care settings [34, 45]. Further exploration of these perceptions among clinicians in U.S. CMHAs is needed, as such beliefs may represent barriers to successful implementation or may reflect the absence of a coordinated multidisciplinary approach indicated for a high-need patient population.

### Limitations

Although a strength of this evaluation is the fact that agencies that participated represent the population of CBTp-SC agencies in the U.S., generalizability may be hampered by the fact that our sample of trained practitioners and administrators is small and disproportionately female and white. A related limitation was the omission of race and ethnicity items in the Phase 1 survey, constraining our ability to characterize our sample in relation to the population of CMHA practitioners. Although our response rate is consistent with the estimated 52.7% mean response rate observed in organizational research [46], our final sample represents 42% of the clinicians who had been trained in CBTp-SC. The response rate was impacted by a host factors, including those known (e.g., 27% of trainees’ organizational email accounts were no longer active) and those suspected (e.g., shifts in roles and responsibilities, increased workload and stress related to the beginning of the COVID-19 pandemic). In addition, we cannot discount the possibility that sampling bias may have affected our findings, such that practitioners who held less favorable views about CBTp-SC did not elect to participate or were no longer a member of the sampling frame due to discontinuing their interactions with the CBTp implementation team. We attempted to mitigate this possibility through our outreach to participants who had dropped out of the training program prematurely and through language soliciting input among both active and inactive CBTp-trained practitioners. Similarly, we highlight the potential for allegiance bias in our evaluation of these preliminary implementation outcomes, which we attempted to minimize by ensuring that the implementation lead and practice facilitators abstained from data collection. Because our sample of CBTp-SC was small, we are unable to assess facilitators and barriers to CBTp-SC allegiance beyond those that were identified by our stakeholder interviews. We did not ascertain the extent to which stepped care decision-making for determining level of care is occurring systematically–as prescribed by the CBTp-SC framework [6]. This is an important area of exploration for future research and an area of consideration for organizations adopting a stepped care approach. Furthermore, although our Phase 1 respondents indicated high average penetration across their cumulative caseload, with most (80.4%) continuing to administer the intervention at the follow-up timepoint, there was substantial variability in the number of clients treated with CBTp among individual respondents. Due to the resource intensity of training practitioners in an evidence-based psychotherapeutic intervention and the high need among clients, organizations should develop internal processes to ensure that trained clinicians are supported in delivering EBTs. Finally, because focus groups and interviews were held just as pandemic risk mitigation measures were being put in place, we were unable to conduct focus groups with CBTp-SC service users, and therefore do not yet know how the model is perceived by its intended beneficiaries.

## Conclusions

Future research should further explore the feasibility of implementing CBTp-SC in community behavioral health settings; the extent to which CBTp-SC uptake and sustainment is impacted by individual and organizational-level variables; and the perceptions and clinical and functional outcomes of CBTp-SC patients. As this is the first study evaluating the theoretical assumptions and implementation outcomes of CBTp-SC, additional research is needed to explore cost, fidelity, service utilization, optimal model delivery methods, and optimal settings for a stepped care approach. Objective metrics of efficiency (e.g., wait times for a CBTp clinician) and effectiveness (e.g., clinical outcome data) are needed to better understand the costs and benefits of CBTp-SC implementation compared to services as usual and to a non-stepped care approach to CBTp implementation.

## Data Availability

The datasets used and/or analyzed during the current study are available by request from the corresponding author.

## Abbreviations

AIM: Acceptability of Intervention Measure
ANOVA: Analysis of Variance
CBTp: Cognitive Behavioral Therapy for psychosis
CBTp-SC: Cognitive Behavioral Therapy for psychosis Stepped Care
CMHA: Community Mental Health Agency
FIM: Feasibility of Intervention Measure
IAM: Intervention Appropriateness Measure
IAPT: Improving Access to Psychological Therapies
LI-CBTp: Low-Intensity Cognitive Behavioral Techniques for psychosis
RCT: Randomized Controlled Trial
REDCap: Research Electronic Data Capture
SMI: Serious Mental Illness
